# The Relationship Between Experiences of Daily Events and Sleep Duration in Adulthood

**DOI:** 10.1093/geroni/igaa057.2174

**Published:** 2020-12-16

**Authors:** Sun Ah Lee, Susanna Joo, Hye Won Chai, Hey Jung Jun, David Almeida

**Affiliations:** 1 Yonsei University, Seoul, Republic of Korea; 2 The Pennsylvania State University, University Park, Pennsylvania, United States

## Abstract

This study aimed to examine how stressors and positive events are related to sleep duration in daily life and whether these associations differed by age. The second wave of National Study of Daily Experiences of Midlife in the United States study was used (N=1,851). Reports of daily events was coded as two categorical variables indicating experiences of concurrent and previous-day daily events: experiencing both stressors and positive events, only stressors, only positive events, and neither (reference). Results from multilevel analysis showed that experiencing an event, either a stressor or a positive event, was associated with shorter amount of sleep the same day compared to a non-event day. In particular, sleep duration was shorter when individuals experienced stressors compared to when they only reported positive events the same day. There were no age differences in these associations. Findings suggest that stressors exert a stronger influence on daily sleep than positive events.

